# Marrow Hematopoietic Stem Cells Revisited: They Exist in a Continuum and are Not Defined by Standard Purification Approaches; Then There are the Microvesicles

**DOI:** 10.3389/fonc.2014.00056

**Published:** 2014-04-04

**Authors:** Peter J. Quesenberry, Laura Goldberg, Jason Aliotta, Mark Dooner

**Affiliations:** ^1^Department of Medicine, Division of Hematology/Oncology, Rhode Island Hospital, Providence, RI, USA

**Keywords:** stem cell, cell cycle, stem cell purification, vesicles, circadian rhythm

## Abstract

Current concepts of hematopoiesis are encompassed in a hierarchical stem cell model. This developed initially from studies of colony-forming unit spleen and *in vitro* progenitors for different cell lineages, but then evolved into a comprehensive model of cells with different *in vivo* differentiative and proliferative potential. These cells were characterized and purified based largely on expression of a variety of lineage-specific and stem cell-specific surface epitopes. Monoclonal antibodies were bound to these epitopes and then used to physically and fluorescently separate different classes of these cells. The gold standard for the most primitive marrow stem cells was long-term multilineage repopulation and renewal in lethally irradiated mice. Progressive work seemed to have clonally defined a Lineage negative (Lin−), Sca-1+, c-kit+, CD150+ stem cell with great proliferative, differentiative, and renewal potential. This cell was stable and in the G0 phase of cell cycle. However, continued work in our laboratory indicated that the engraftment, differentiation, homing, and gene expression phenotype of the murine marrow stem cells continuously and reversibly changes with passage through cell cycle. Most recently, using cycle-defining supravital dyes and fluorescent-activated cell sorting and S-phase-specific tritiated thymidine suicide, we have established that the long-term repopulating hematopoietic stem cell is a rapidly proliferating, and thus a continually changing cell; as a corollary it cannot be purified or defined on a clonal single cell basis. Further *in vivo* studies employing injected and ingested 5-Bromodeoxyuridine (BrdU), showed that the G0 Lin-Sca-1, c-kit+ Flt3− cell was rapidly passing through cell cycle. These data are explained by considering the separative process: the proliferating stem cells are eliminated through the selective separations leaving non-representative dormant G0 stem cells. In other words, they throw out the real stem cells with the purification. This system, where the marrow stem cell continuously and reversibly changes with obligate cell cycle transit, is further complicated by the consideration of the impact of tissue microvesicles on the cell phenotypes. Tissue microvesicles have been found to alter the phenotype of marrow cells, possibly explaining the observations of “stem cell plasticity.” These alterations, short-term, are due to transfer of originator cell mRNA and as yet undefined transcription factors. Long-term phenotype change is due to transcriptional modulation; a stable epigenetic change. Thus, the stem cell system is characterized by continuous cycle and microvesicle-related change. The challenge of the future is to define the stem cell population.

## Introduction

### Notes on cell cycle and cell phenotype

**Figure 1:**
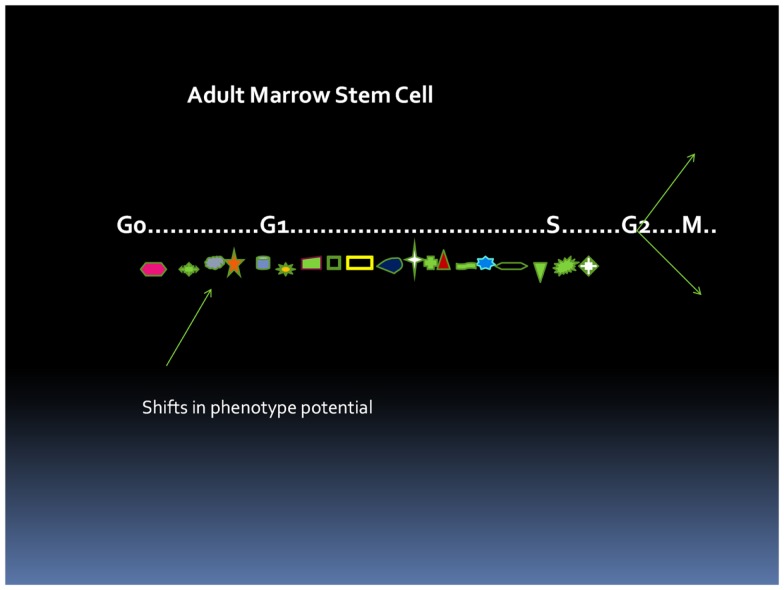
**Cell cycle-related changes in phenotype**. At each point in cycle transit, the stem cell has a different phenotype. This presumably could reverse with an asymmetric division.

The cell cycle status of a stem cell is a major determinant of cell phenotype and potential. A stem cell progressing through cell cycle will be continually changing its phenotype as to surface epitopes, RNA and DNA content, metabolic status, and overall potential and thus cannot be precisely characterized as a single entity (Figure 1).

The G0 state may be characterized by 2N DNA and low RNA levels with G1 showing increasing RNA levels and S showing increasing DNA levels. Mitosis then starts the whole sequence over with the three conceptual outcomes: (1) a symmetric division in which each daughter cell retains its identity, (2) a symmetric division in which each daughter cell has differentiated, and (3) an asymmetric division in which one daughter cell has differentiated and in which the other has maintained its original identity. There is also the possibility of cell death of one or both daughters. In general, it is assumed that the final end result of divisions in the stem cell population should maintain the stem and differentiated populations on a steady state basis. An excess of symmetric divisions of stem cells would result in leukemia and an excess of differentiated end results would result in exhaustion and aplastic anemia. These considerations are paramount to understanding current marrow stem cell biology.

### The first clonal stem cell – the colony-forming unit spleen

**Figure 2:**
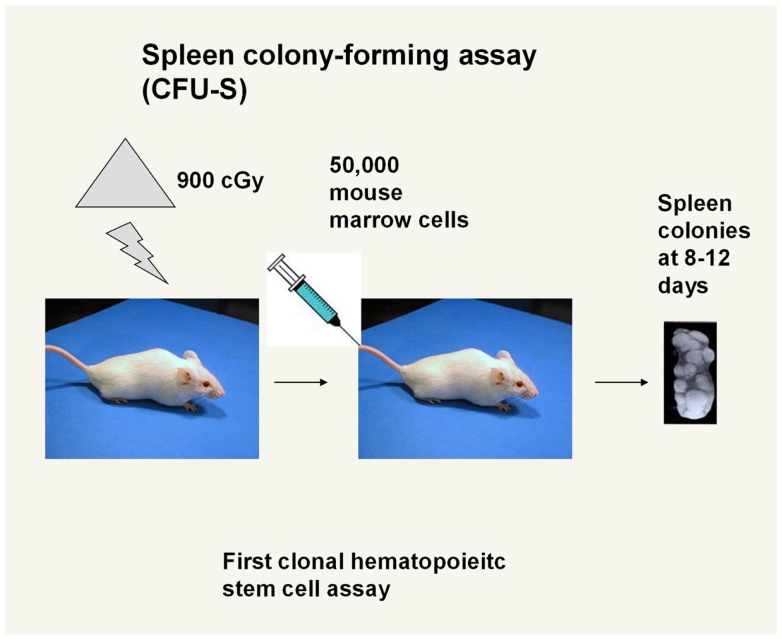
**Spleen colony-forming assay**. Marrow cells are injected into an irradiated mouse and single cell-derived clones appear as “lumps” on the surface of the spleen. Myeloid, erythroid, and megakaryocyte cells were seen in the lumps in various mixtures.

The colony-forming unit spleen (CFU-S), as reported by Till and McCulloch (1), was the first description of a clonal stem cell unit. They received the Lasker award in 2005 for this seminal work on stem cells. While there has been a long period when this assay has been regarded as not defining the true stem cell, our own work would suggest that it indeed is a very good stem cell assay. Many of the original insights on the CFU-S would appear to be valid, in light of current work on adult marrow stem cells. The assay itself involves injecting murine marrow cells intravenously into lethally irradiated mice and then counting lumps (or clones) on the spleen which were stained with Bouin’s fixative (Figure 2).

The cellular makeup of the colonies varied depending upon the location in the spleen, but these cells had the potential for differentiation into all myeloid cell classes and for self renewal. The CFU-S was characterized as a cell with an extensive capacity for differentiation and proliferation along with self renewal. It was shown that bumps on the spleen were clonal and that cells from a colony could form colonies in secondary irradiated hosts. Thus, the characteristics of the marrow stem cells were outlined as a cell which had extensive proliferative and differentiative potential into marrow myeloid cell types and which could self renew. An important feature of these early studies was observations of the heterogeneity of the formed colonies as to size, cell number, cell type, and location (2). Furthermore, it appeared that different cells might be monitored if the colonies were counted at 9, 12, or 14 days with more primitive cells being monitored by colonies, which arose at longer time intervals (3). Perhaps the most striking and important feature of the CFU-S was the total heterogeneity of self renewal from individual colonies. This would appear to be particularly relevant to our current concepts of the biology of adult marrow hematopoietic stem cells. In discussing how this lax regulation (heterogeneity) could be reconciled with the orderly behavior of normal hematopoietic tissue, Till et al. (2) drew an analogy with radioactive atoms, “If one studies a large number of radioactive atoms, one sees a very regular pattern of decay following an exponential law. However, if one studies individual atoms, they are found to decay in an unpredictable fashion at random. It appears possible that our studies of the progeny of single cells display the random feature of hematopoietic function, while study of large populations of cells reveals the orderly behavior of the whole system. From this point of view, it is the population as a whole that is regulated rather than individual cells and it is suggested that control mechanisms act by varying the “birth” and “death” probabilities.” As we will develop below, these are very prescient comments, which apply to the current state of stem cell biology.

### The progenitor era – it fit so well

**Figure 3:**
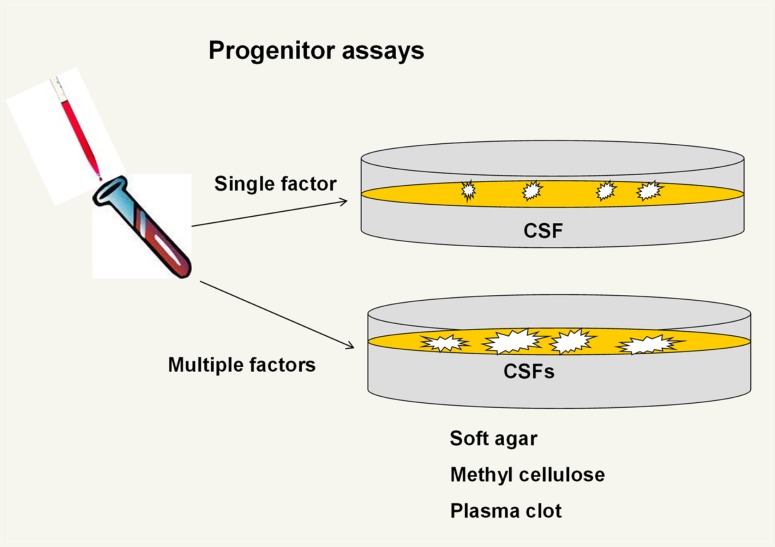
**Progenitor assays**. Initial assays were for granulocyte– macrophage colony units but then a variety of single factor and then multiple factor clonal units were described. In general, the multifactor responsive progenitors formed larger colonies.

The next thrust of research in the stem cell field was the definition of progenitor classes of hematopoietic cells. Bradley and Metcalf (4) and Pluznik and Sachs (5) described the *in vitro* cloning in semisolid media of marrow cells that form granulocyte–macrophage colonies. As work here developed, the systems involved various semisolid matrices including soft agar, methyl cellulose, and plasma clot and various sources of “colony-stimulating factors” including mouse embryo-conditioned media, serum from endotoxin-treated mice, and cell feeder layers (Figure 3).

**Figure 4:**
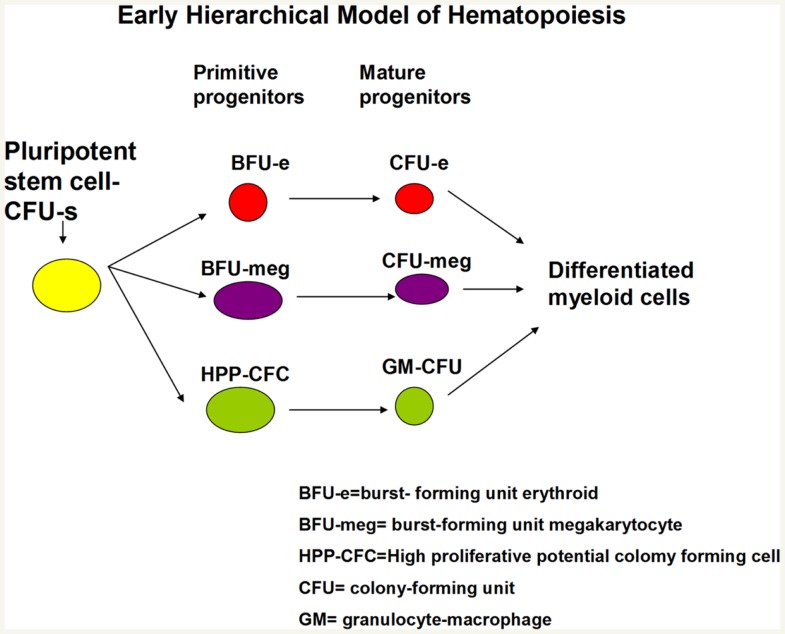
**Hierarchical model of hematopoiesis pluripotent stem cells give rise to progenitors with progressively less proliferative and renewal potential and more differentiated characteristics**.

This work expanded as different investigators described cells giving rise to erythroid and megakaryocyte colonies (6) and then subsets of these lineage-specific colonies were described such that large colonies responding to multiple growth factors were termed burst-forming unit erythroid (7) and burst-forming unit megakaryocyte (8), while smaller colonies responding to one or a few cytokines were termed colony-forming unit erythroid or megakaryocyte. Relatively primitive cells giving rise to blast colonies (9) or high-proliferative potential colonies (10) were then defined and felt to possibly be surrogates for long-term repopulating marrow stem cells. Dr. Ogawa described a bewildering array of different colony types with from one to five lineages arising from single cells. Almost all possible combinations of differentiated cell colonies were seen (4). This gave rise to a hierarchical model with the multipotent CFU-S giving rise to multipotent progenitors (MPPs) with more limited potential which then, in turn, gave rise to bi or unipotent progenitors followed by recognizable differentiated myeloid cells. A simplified early hierarchical model is presented in Figure 4.

This suggested a very orderly system of hematopoiesis regulated by a series of cytokines or colony-stimulating factors with more primitive cells needing more factors to express their phenotype. Dr. Ogawa also published data showing that within one cell cycle transit from a blast colony-forming cell, totally different lineages could be pursued by the daughter cells (4). Thus, one daughter might give rise to a granulocyte–macrophage colony while the other daughter gave rise to an erythroid–megakaryocyte colony. The implications of these careful observations were generally ignored. These data were akin to throwing a bomb in the middle of any hierarchical model. As we will develop below, these data fit an alternative continuum model of hematopoiesis. With the definition of many progenitor cell classes, the emphasis of research turned to the precise clonal definition of the “true” long-term repopulating marrow stem cells and a full elucidation of the complex hematopoietic hierarchy.

## The Purificators

**Figure 5:**
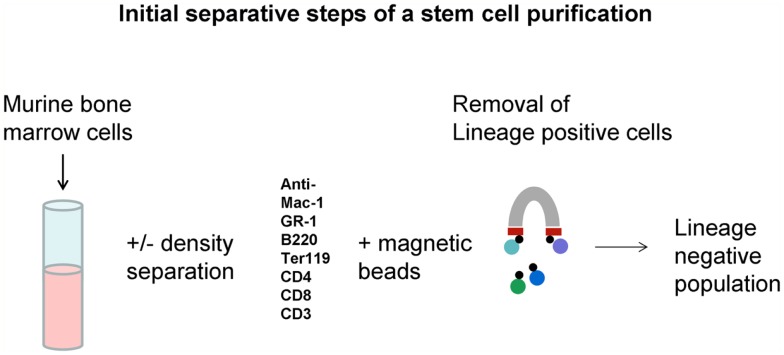
**Lineage negative cell population**. Mouse marrow is depleted of cells expressing markers characterizing differentiated marrow cells using a magnetic-based cell removal.

**Figure 6:**
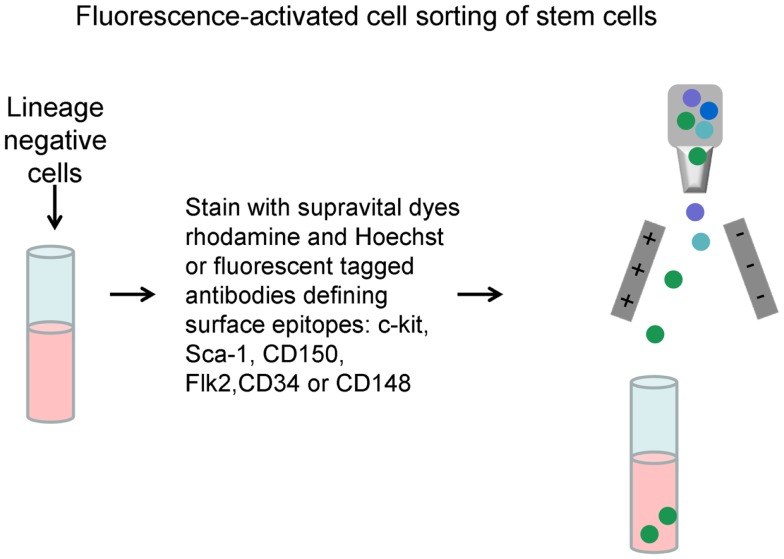
**Long-term hematopoietic stem cell (LT-HSC) separation**. Lineage negative cells are labeled with either supravital dyes or “stem cell” antibodies and then separated by fluorescence-activated cell sorting.

Early work suggested that cells with markers of differentiation had low to no long-term repopulating cells as defined by long-term multilineage repopulation in a lethally irradiated mouse. These studies were usually carried out using congenic mouse transplant models, the CD54.1 and CD45.2 strains being most often employed. They also frequently extended to secondary repopulation in irradiated host to demonstrate “renewal.” Typically antibodies to differentiated cell markers with iron tags were incubated with marrow cells and positive “differentiated” cells removed by magnetic adherence. Then, this lineage negative population was incubated with antibodies to cell surface epitopes, the presence or absence of which, enriched for long-term repopulating cells (5). Many candidate stem cell markers were evaluated with positivity for c-kit, Sca-1, intermediate staining for Thy.1, and negativity for FLK2 (11–13) being initially defined markers and CD150 or Slam (14) and CD34 (15) also currently in vogue for definition of the stem cell. These studies showed a lack of tight correlation with the purified stem cells and CFU-S and led to the dismissal of CFU-S as a relevant stem cell; as we will develop below, this was probably a fundamental mistake. These studies also led to the evolved dogma that a stem cell could only be defined clonally. The general aspects of a stem cell separation are pictured in Figures 5 and 6.

Studies continued to focus on the “holy grail” of stem cell biology; the characterization and isolation of the long-term multilineage repopulating stem cell which had to be defined on a clonal basis. The continued evaluation of transplant potential of cells separated by expression of various surface markers led to a beautiful and rationale model of hematopoiesis. At one point, I think everyone, including myself, was an unrepentant purificator.

**Figure 7:**
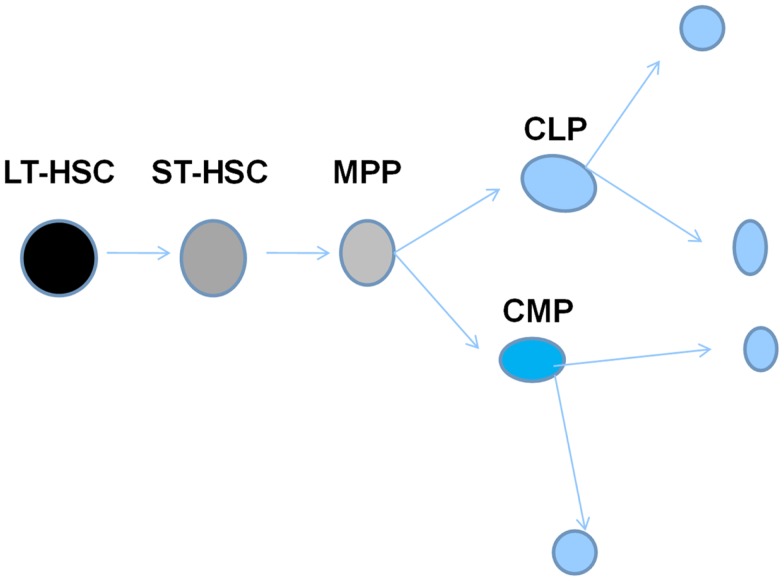
**Hierarchical model of marrow stem cell biology**. This depicts a system with an orderly progression from cells with high-proliferative and renewal potential to cells with a progressive loss of such potential, but a gain of differentiated features.

Murine marrow cells were labeled with antibodies to different cell surface proteins, separated by FACS and then assessed for long and short-term engraftment and for the lineage choice after engraftment. Long-term hematopoietic stem cells (LT-HSCs) were separated on the basis of lineage negative status and expression of the surface epitopes c-kit and Sca-1 with either Thy 1.1 expression or absence of FLK2. Cells with a multilineage repopulation potential not exceeding 6–8 weeks were then characterized by gain of FLK2 expression. Loss of Thy 1.1 expression with full expression of FLK2 characterized the next differentiation step to the MPP. Common lymphoid and myeloid stem/progenitor cells were then defined by selective expression of IL-7, Fcr receptor 11/111, and CD34. This created an elegant model of hematopoiesis as outlined in Figure 7.

CD150 was subsequently added as a further definer of LT-HSC. With these separations, a very small number of LT-HSC could repopulate an irradiated host mouse. The holy grail appeared to be within reach and this is pretty much the standard hematopoiesis model today.

### An alternative model of hematopoiesis or the continuum heresy

In early studies on engraftment into non-myeloablated mice, murine marrow cells were treated with the cytokines IL-3, IL-6, IL-11 and steel factor in an attempt to increase engraftment levels. However, after 48 h of culture, there was a marked decrease in engraftment capacity (16, 17). Subsequent studies showed, however, that the loss of engraftment was temporary (18) in six separate experiments engraftment returned to at or above baseline levels with further culture. This was inconsistent with a hierarchical model and suggested a continuum of changing potential. Subsequently, using either whole unseparated murine marrow or highly purified murine marrow Lin−, rhodamine low, Hoechst low (LRH) stem cells or Lin− Sca-1+ cells driven through cell cycle by exposure to cytokines, either IL-3, IL-6, IL-11, and steel factor or thrombopoietin, Flt3, and steel factor, we demonstrated that different phenotypic stem cell characteristics were apparent at different points in cycle or times in culture. These changes varied and were generally reversible. Mapping purified stem cells with propidium iodide as they progressed through a cytokine-stimulated cell cycle transit allowed us to estimate phases of cycle for these experiments. We investigated short- and long-term engraftment (18, 19), progenitor numbers (20), homing to marrow (21) and lung (22), expression of adhesion proteins (23, 24) and cell cycle receptors, stem cell surface markers, and cell cycle and other transcriptional regulators (25–27). All were found to vary with cycle transit but at different points in cycle. Further studies showed that differentiation into megakaryocytes and granulocytes occurred at specific cycle times, so-called “hotspots” and these were reversible (28). Formation of epithelial lung cells from engrafted marrow also varied with cell cycle (22). Most recently, we have shown that microvesicle entry into marrow stem cells varies with cell cycle status (29). This is probably another determinant of stem cell fate. Essentially, every biologic parameter which we investigated varied as stem cells progressed through cell cycle under cytokine stimulation. Data on phenotype variation after engraftment into irradiated mice are presented in Table 1.

**Table 1:** **Reversible variations in marrow cell phenotype with cell cycle phase after engraftment into lethally irradiated mice**.

Characteristic	Whole marrow	LRH	Lin-Sca-1+
Engraftment	Nadir late S/early G2	Nadir late S/early G2 first and second cycles with recovery in between	
Homing			Nadir late S/G2 second cycle
Increase in lung conversion			Early S-phase
Differentiation into megakaryocyte and proliferative granulocytes		Around G1/S interface	
Differentiation into non-proliferative granulocytes		Late S-phase	

Cytokine cocktails were either: IL-3, IL-6, IL-11, and steel factor or FLT3L, steel factor, and thrombopoietin. Cycle mapping with cytokine-stimulated (IL-3, IL-6, IL-11, and steel factor) purified LRH stem cells showed an initial cycle length of 36–40 h with subsequent cycle occurring every 12 h. G1 phase was estimated at about 18 h and mid-S-phase at about 28–30 h. Studies of expression of over 40 different genes showed low-level expression of all in LRH cells at isolation and a relatively chaotic variation of expression with cycle transit (25). Adhesion proteins vary but generally drop with cycle transit; CD44 and alpha L increased at 48 h. Studying Lin-Sca-1+ marrow cells during cycle transit under IL-3, IL-6, IL-11, and steel factor stimulation, expression of CD34, CD45R, c-kit, Gata-1, Gata-2 Ikaros, and Fog were stable while Sca-1, Mac-1, c-fms, c-mpl, Tal-1, endoglin, and CD4 showed variation in expression. All showed reversibility except Tal-1, endoglin, and c-mpl. We have also studied LRH cells stimulated by different combinations of cytokines and cloned on a single cell basis (30). Mean cloning efficiency was 31.7% with a range of 8.3–65%. Gross colony morphology and size showed total heterogeneity. Over 100,000 cells per clone were seen at the highest cytokine level. Virtually total heterogeneity as to differentiation phenotype at different points in cycle (0, 18, 32, 40, and 48 h culture) was also demonstrated. There were, however, different patterns of differentiation at different points in cycle; again total individual cellular heterogeneity with population profiles.

**Figure 8:**
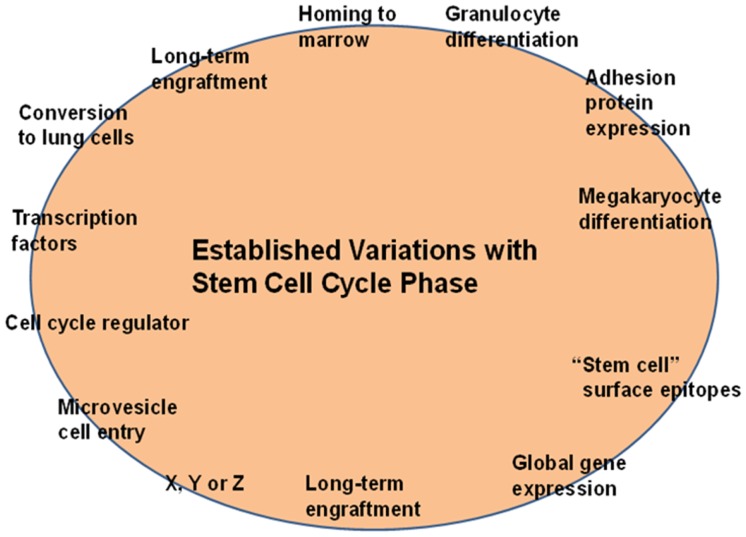
**Cell cycle phenotype variation**. As the stem cell progresses through cell cycle (the circle), different potentials exist which are expressed only if the cell is appropriately interrogated at that point in cell cycle. The interrogations or stimulations may consist of *in vivo* engraftment and microenvironment exposure, cytokine or growth factor exposure, or microvesicle exposure (see below).

**Figure 9:**
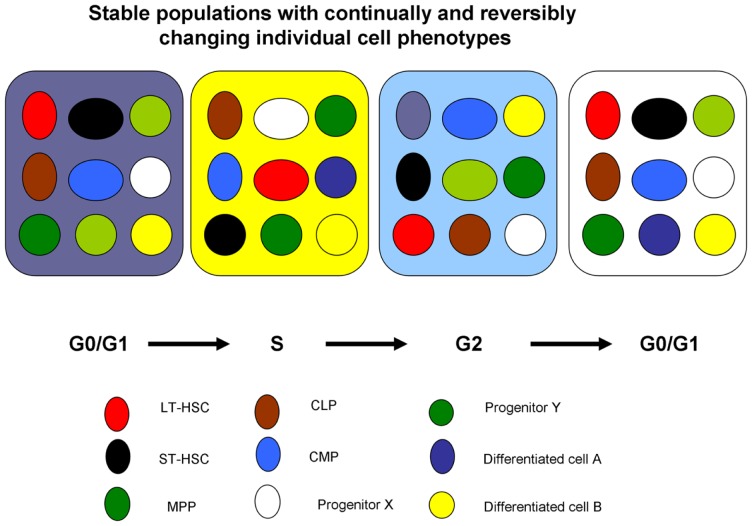
**A population model of stem cell biology**. Each colored circle represents an individual cell at a certain point in cell cycle. As the cell progresses through cycle (different colored boxes), its potential changes but then returns to its original potential. For instance, the red circle in box G0/G1 has LT-HSC potential if infused into an irradiated mouse, but later when the same cell is in S-phase, a brown circle, its potential is that of a common lymphoid progenitor and then when in G2 that of differentiated cell A. It returns to its original potential when in the next G0/G1. These are all potentials and nothing happens if there is not an appropriate interrogation.

These observations, that there were cycle-related reversible changes in stem cell phenotype, suggested a continually changing population of cells consistent with a continuum of cellular potential related to cell cycle phase. This further suggested that while there might be a stable stem cell population, the individual cells or entities in the population were continually changing – shades of Till et al. (2) (see above). A simplified continuum model is presented in Figure 8 and a more complex model in Figure 9.

This model is essentially a model of cell cycle-related continuous change of potentials. It implies that there always will be cohorts of the marrow stem/progenitor population available to respond to any relevant need and the different populations of cells will be continually entering into a responsive window. This is not consistent with the standard hierarchical model of stem cell biology.

## Cell Cycle Status of Long-Term Engrafting Multilineage Marrow Stem Cell – “Pay No Attention to That Man Behind the Curtain”

A critical consideration as to the biologic relevance of our observations on phenotype change in stem cells as they progress through a cytokine-stimulated cell cycle transit was whether *in vivo* the marrow stem cell is a cycling cell. Much current dogma has it that it is a dormant non-cycling cell, but as we will show, the foundation for this conclusion may have been based on studying the wrong cell population; the purified LT-HSC. A great deal of mechanistic research has now occurred focusing on the purified LT-HSC. Studies have implicated a myriad of entities as key regulators of hematopoiesis. There have also been a large number of studies attempting to define the hematopoietic stem cell niche employing purified stem cells as a critical tool. However, certain disquieting observations were ignored or not put in the proper context. As noted above, the Ogawa cycle studies (31, 32) indicated that there could not be a straightforward hierarchy, then there were the observations on stem cell plasticity. Much time and effort was wasted here on disputing transdifferentiation versus dedifferentiation, when in fact these studies simply demonstrated that hematopoietic marrow stem cells could be induced to differentiate into non-hematopoietic-cell classes. This of course did not fit with the hematopoietic hierarchy at all and engendered some vigorous attacks from those espousing the conventional hierarchical dogma. Demands that the studies show robustness, be clonal, and not be due to fusion were essentially ignoratio elenchi or red herrings (33). Another disquieting fact was ignored. During these separations, the great bulk of marrow long-term repopulating stem cells are lost and the losses are not random. In a non-ablated transplant model, the loss of engraftable stem cells with a LRH purification ranged from 93.6 to 99.2% of what was present in the starting marrow population (34). We have now confirmed these losses in a lethally irradiated mouse model. A critical consideration is that while the final product of purification gives highly purified cells with a specific functional characteristic, the bulk of the stem cells are in the discard fractions. In these fractions, while the percent of stem cells is low, the total number of stem cells is vastly superior to the number seen in the purified fractions.

## Stem Cell Purification and the Holy Grail of Single Cell Clonality – A Research Field Misled

If one enters the descriptor “murine hematopoietic marrow stem cells” into PubMed, one gets over 17,000 hits. Many are not applicable to adult murine marrow stem cells as classically defined; rather they refer to human studies, mesenchymal stem cells, aspects of stem cell plasticity, or other unrelated topics. However, screening these “hits,” there were a large number which referred to aspects of murine adult stem cell biology. These studies involved different purified populations of stem cells as outlined above. In general, the initially published surface phenotype of a functionally defined cell was assumed to hold and functional studies were only rarely carried out. It was assumed that the surface epitope phenotype represented a specific class of stem cells with specific functional characteristics such as long-term or short-term multilineage engraftment or engraftment with differentiation into lymphoid cells. Thus, the vast majority of reported studies of stem cell characteristics: gene expression, cytokine responsiveness, transcriptional regulation, homing, niches, engraftment, and cell cycle status were carried out employing these surrogate phenotypes and assuming stability of these phenotypes. Our continuum studies challenge these concepts. In a similar vein, we have recently reported that the short-term hematopoietic stem cell (ST-HSC, as defined by Lin−/Sca-1+/c-kit+/Flk2−) was not short-term in our functional experiments involving studies of stem cell homing (35). In addition, as noted above, in studies on highly purified LRH stem cells, isolated at different points in cell cycle, and grown as single cells in a permissive cytokine cocktail, total heterogeneity of differentiation phenotype was demonstrated (30). Thus, the phenotype was varied and no stability could be inferred. This harks back to the isotope model of Till et al. (2).

We were not the only ones to publish data challenging the conventional concepts of a hierarchical system. Sieburg and colleagues (36) studied 97 individual HSCs in long-term transplantation assays. HSC clones were obtained from unseparated bone marrow (BM) through limiting dilution approaches. Following transplantation into individual hosts, donor-type cells in blood were measured bimonthly and the resulting repopulation kinetics were grouped according to overall shape. Only 16 types of repopulation kinetics were found among the HSC clones even though combinatorially 54 groups were possible. These data were also inconsistent with a straightforward hierarchy. As pointed out to authors in a published correspondence (37), one needs to alter only a few parameters to arrive at the existence of a huge number of stem cell phenotypes which would be most consistent with a continuum model of stem cell biology. Even single cell repopulation assays with highly purified stem cells have been inconsistent with defined stem cells giving rise to an ordered hierarchical system of hematopoiesis. The capacity to isolate a specific stem cell phenotype is of course dependent upon the stability of that phenotype. This in turn is dependent upon the quiescence of the cell under consideration. A cycling cell continually changes phenotype, as we have repeatedly demonstrated, and thus cannot be characterized by a single set of cell surface characteristics. Stability was addressed in more general terms by Montaigne in “Of Repentance” where he states “all things in it are in constant motion; the earth, the rocks of the Caucasus, the pyramids of Egypt, both with the common motion and with their own. Stability itself is nothing but a more languid motion.” These considerations led to a detailed evaluation of the cell cycle status of the murine engrafting marrow stem cell.

## The Cell Cycle Status of Stem Cells – They are Cycling!

**Figure 10:**
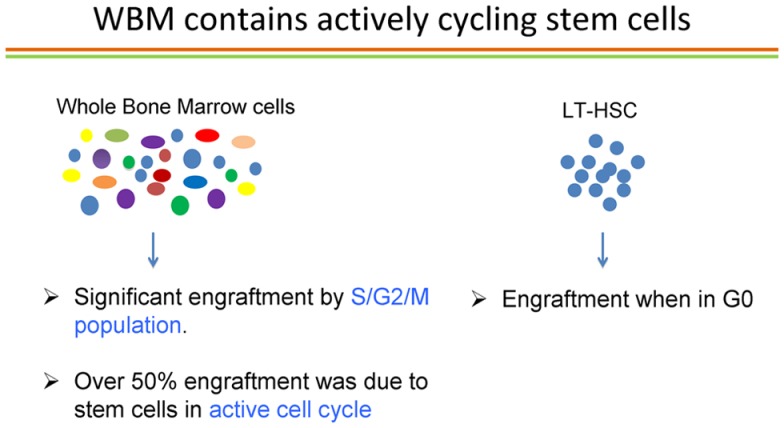
**Cell cycle status of long-term repopulating cells in whole marrow and in the LT-HSC purified stem cell population**. Using either a Pyronin/Hoechst (S/G2/M) or tritiated thymidine suicide (S-phase) approach, and evaluating whole unseparated murine marrow cells, from over 50 to 80% of the cells were in cell cycle. When purified LT-HSC was evaluated, almost all the LT-HSC was in G0.

Passegue and colleagues (38) published elegant studies showing that the long-term repopulating stem cell characterized as Lin−, c-kit+ Sca-1+ Flk2− only engrafted as a G0 cell. If this was the status of the true marrow stem cells, then our studies of cycle transitioning stem cells could represent an *in vitro* artifact of the culture systems employed. Accordingly, we embarked on a detailed evaluation of the cell cycle status of LT-HSC. With a few outliers, we essentially confirmed the prior studies by Passegue et al. (38) on purified marrow stem cells. We purified LT-HSC into G0, G1, and S/G2/M phases using the supravital dyes Pyronin and Hoechst and then competitively engrafted them into lethally irradiated mice. With a few rare events, essentially all engraftment was found to reside in the G0 compartment of LT-HSC. However, in the review of the literature noted above, we found that essentially all cell cycle studies of engraftable stem cells had been carried out on highly purified stem cells, not on the unseparated whole marrow population. We sought to remedy this oversight by studying unseparated murine marrow cells and determining the cell cycle status of long-term engrafting cells in these cell populations. Accordingly, we separated murine marrow cells into G0, G1, or S/G2/M populations and then determined long-term multilineage engraftment and secondary engraftment. Over 50% of engraftment was found in the S/G2/M populations of marrow cells. This represented an instantaneous view of cycle status of stem cells and implied that virtually all stem cells must be in cycle (39). We sought to confirm these observations with an alternative method of cycle determination; tritiated thymidine suicide. In this approach, high specific activity tritiated thymidine, a beta emitter, is incubated with marrow cells and, if the cells are synthesizing DNA, the thymidine will be incorporated into the cellular DNA and the cell will then die a radioactive death. The beta particles only exert their activity within the cell and there is no innocent bystander effect. The control marrow cells are incubated with a comparable amount of cold thymidine. At the end of 30 min, a large excess of unlabeled thymidine is added, which inhibits further uptake of the radiolabeled thymidine and the washed cells are then evaluated for long-term multilineage engraftment in a competitive transplant model in lethally irradiated mice. The decrease in engraftment of the tritiated thymidine-treated cells compared to the unlabeled thymidine-treated cells then represents the cell cycle status of these cells. Applying this approach to unseparated B6.SJL marrow cells and then competitively transplanting them into lethally irradiated C57BL/6J mice, we demonstrated that over 70% of the cells had passed through S-phase, thus confirming the active cell cycle status of long-term engrafting cells in normal unseparated murine marrow cells (39). This is summarized in Figure 10.

**Figure 11:**
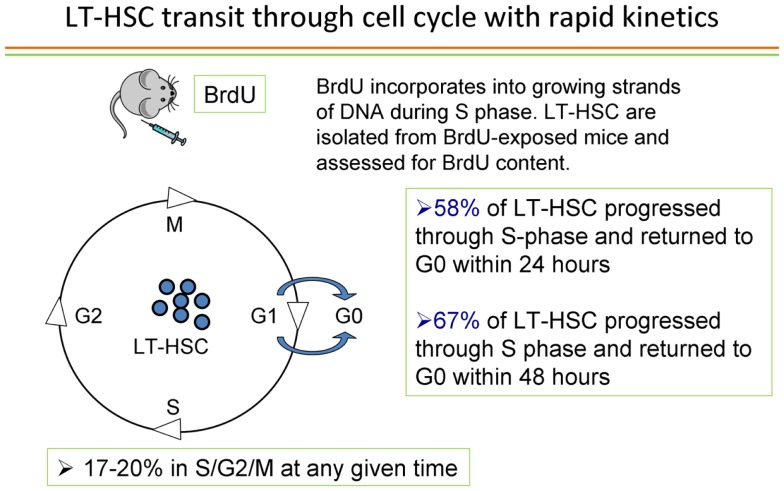
***In vivo* cell cycle transit of G0 LT-HSC – an individual experiment**. The G0 LT-HSC is rapidly passing through cell cycle*in vivo*.

These observations suggested that almost all long-term repopulating stem cells in murine marrow are in cycle. What about the G0 status of engrafting purified LT-HSC? What is the history of these cells? Are they passing through cycle or do they represent a rare permanently quiescent population of cells? In order to address these questions, we utilized *in vivo* BrdU labeling of marrow cells over time. BrdU was administered to B6.SJL mice intraperitoneally (1 mg every 8 h) over 48 h along with BrdU in the drinking water and at different time points, G0 LT-HSC were interrogated for BrdU labeling. BrdU is incorporated into cells synthesizing DNA and thus provides a cycle passage history for the G0 LT-HSC. At 24 h, 58% of G0 LT-HSC was labeled and at 48 h over 65% were labeled (39). The method and results of a representative experiment is shown in Figure 11.

Additional studies ruled out BrdU activation of stem cells into cell cycle. Thus the engraftable LT-HSC is continuously and rapidly transiting cell cycle. This has profound implications to the interpretation of stem cell studies since it indicates that the stem cell phenotype must be continually changing and thus purification of the stem cells is not feasible, rather definition of the stem cell population is the critical issue.

## Why the Difference in Cycle Status between Purified Stem Cells and Stem Cells in Whole Unseparated Marrow? We Forgot about the Discard!

**Figure 12:**
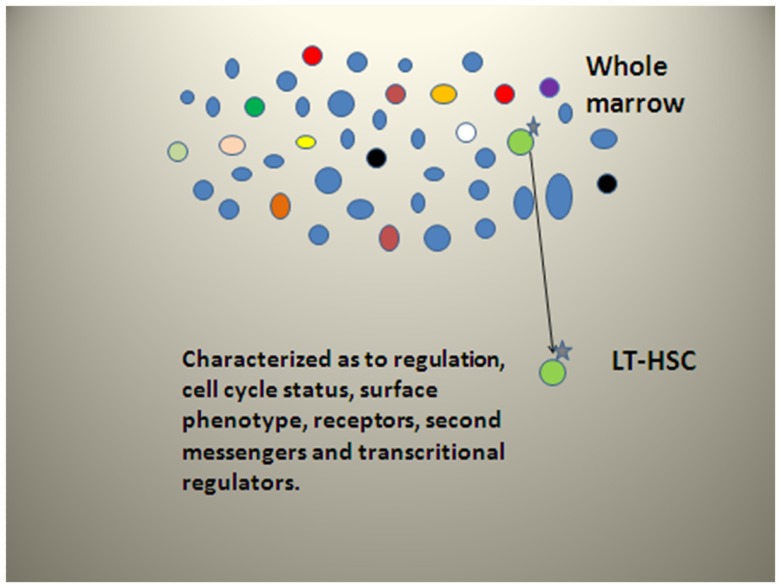
**Stem cell separative strategy for LT-HSC**. Blue cells are differentiated marrow cells while all other colors represent long-term repopulating stem cells in various functional states. The isolated LT-HSC is non-representative.

As noted above, we have published studies on purification of LRH stem cells. We showed then that with purification, from 94 to 99% of stem cell capacity was lost (34). We are just now understanding the true significance of these findings. In the course of stem cell purification, almost all of the long-term engraftable marrow stem cells are discarded. Thus, while the purified cells are certainly enriched in stem-like cells, the discarded populations have almost all the stem cells and these cells are cycling. Our data clearly indicate that the vast majority of long-term repopulating stem cells are lost with the separation, which selects out a non-representative dormant cell with long-term repopulating capacity. Current ongoing experiments indicate that most of the proliferating stem cells are in the lineage positive population. This separative strategy is summarized in Figure 12.

## They all Got Rhythm

All biologic systems have circadian rhythms. They represent basic features of life, but are generally ignored in stem cell studies, because they make stem cell studies very complicated. However, they must be addressed if we are to understand stem cell biology. We have previously carried out relatively limited studies of circadian rhythms of engrafting marrow stem cells and progenitors (40). In these studies, male B6.SJL mice were entrained for 2 weeks in light dark boxes and then marrow harvested at different circadian times [hours after light onset (HALOs)]. We harvested marrow cells at HALOs 4, 8, 12, 16, 20, and 24. C57BL/6J male hosts at HALO 9 were then subjected to 100 cGy whole body irradiation and injected with 40,000,000 marrow cells from each HALO. Engraftment was then assessed in spleen, marrow, and thymus 10 weeks after cell infusion. In studies carried out in July, there were significant nadirs seen at HALO 8 and HALO 24 with up to fivefold differences between comparative peaks. In separate experiments, we determined that host engraftability showed no circadian rhythms for engraftment. There were progenitor nadirs, HPP-CFC, and total progenitors, at 12 and 24 h. Cycle status of HPP-CFC was determined using tritiated thymidine suicide; increased numbers of HPP-CFC in S-phase were seen at 8, 12, and 24 h.

These data introduce another, usually neglected, variable which needs to be addressed in stem cells studies. We are returning to these methods with regard to stem cell cycle studies.

## Stem Cell Plasticity (Ignoratio Elenchi) and Microvesicles

We have outlined extensive plasticity within the hematopoietic stem/progenitor system above. This was variously interpreted and arguments about transdifferentiation, cell fusion, erroneous cell marking, and quantitative and functional significance ensued. A list of criteria for true plasticity was put forward, which included the necessity of the phenomena being “robust,” clonal, functional, and not due to cell fusion. This was commented on in a perspective in science termed “Ignoratio Elenchi or irrelevant conclusions” (33). This controversy served to halt progress in this area of research and the odor of it still lingers. However, there have now been an overwhelming number of studies indicating that after transplantation of marrow cells, many non-hematopoietic cell types evidenced expression of markers of the donor marrow cells. These data were inconsistent with the traditional hierarchical models of hematopoiesis, but fit with the continuum model.

We demonstrated marrow-derived markers in skeletal muscle (41, 42), skin (43), and lung (44) and focused our studies on lung. Utilizing transgenic green-fluorescent protein (GFP) expressing mice as marrow donors, we demonstrated relatively high levels of GFP positive cells in lung, which were further enhanced if host mice were treated with granulocyte colony-stimulating factor after transplantation. Irradiation of host mice was necessary to demonstrate these phenomena.

**Figure 13:**
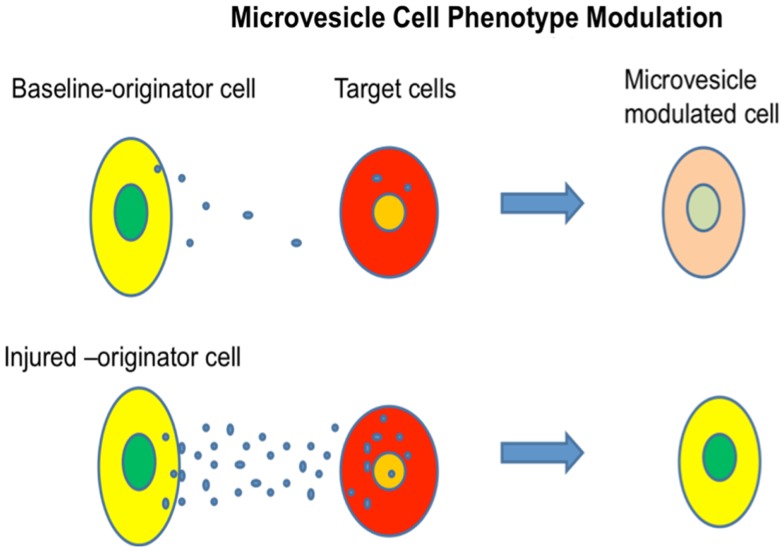
**Microvesicle cell fate modulation**. Cellular-derived vesicles can enter target cells and change their phenotype. If the originator lung tissue is injured with irradiation, the genetic change in target cells is increased.

We investigated mechanisms underlying marrow “transformation” to lung type cells, culturing normal or irradiated lung opposite murine marrow cells, but separated from them by a 0.4 μm cell impermeable membrane, and then determining whether the marrow cells expressed lung-specific mRNA (45, 46). After 2 or 7 days of co-culture, marrow cells expressed high levels of surfactants A, B, C, and D, Clara cell-specific protein, or aquaporin 5 and this expression was significantly higher in marrow cultured across from irradiated lung as compared to non-irradiated lung. Lung-conditioned media could elicit the same genetic changes in marrow cultured in the conditioned media and it was then determined that the active principle could be spun down by ultracentrifugation, the pellet containing large numbers of microvesicles (Figure 13).

**Figure 14:**
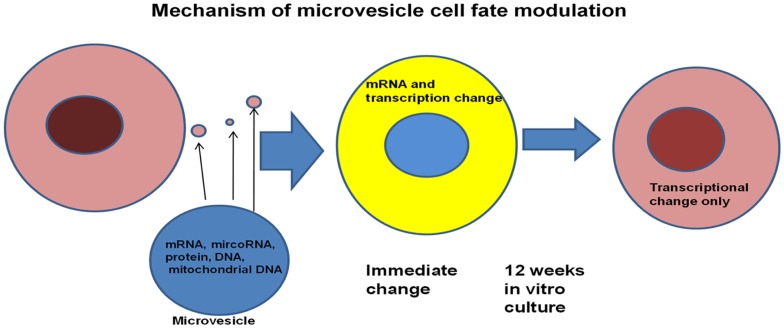
**Genetic changes induced in target cells by microvesicles**. Microvesicles deliver mRNA and a transcriptional activator to target marrow cells. Long lasting changes are due to transcriptional activation of the target cells.

Further studies showed that the genetic change was dependent upon microvesicles entering target marrow cells. All classes of marrow cells imbibed microvesicles and exposure to lung microvesicles were shown to increase the capacity of modulated marrow cells, engrafted into irradiated mice, to “convert” to epithelial lung cells approximately twofold. Microvesicles themselves contained mRNA, protein, microRNA, and mitochondrial and genomic DNA. The microvesicles also expressed many surface proteins including adhesion proteins. Mechanistic studies employing rat/mouse hybrid co-cultures with rat- and mouse-specific primers for mRNA for surfactants B and C showed immediate transfer of originator cell mRNA and also induction of target cell mRNA (46). However, the originator cell mRNA disappeared rapidly with time in cytokine-supported liquid culture, while the target cell mRNA persisted for out to 12 weeks in culture. Transplanted cells also showed expression of lung-specific mRNA out to 6 weeks (as far as tested) in marrow, thymus, liver, and lung. These studies indicated that a persistent epigenetic transcriptional change had occurred (Figure 14).

**Figure 15:**
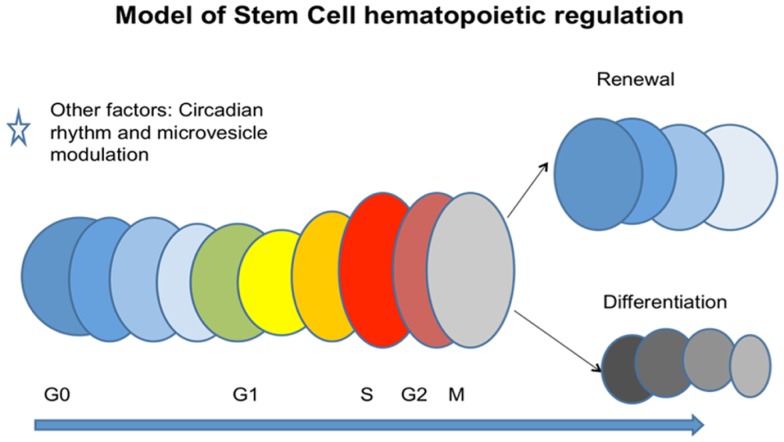
**A cell cycle-based continuum model of stem cell biology**. Stem cells are continually altering phenotype with cycle passage but reversal is seen with an asymmetric division. Microvesicles and circadian rhythm also impact the system to change cell fate.

Very similar studies were carried out with rat liver and mouse marrow co-culture evaluating rat- and mouse-specific mRNA for albumin, with the same results. It was also shown that murine brain and murine heart induced tissue-specific mRNA in target marrow cells. Other work has indicated that mesenchymal-derived microvesicles could mediate *in vivo* repair of renal damage (47). Altogether these data suggest that microvesicles may represent a general biologic cell phenotype modulating mechanism, adding further complexity to marrow stem cell models. A general model encompassing many of the above noted variables is presented in Figure 15.

## To the Last Place of Decimals

There was a time in the late 1800s when physicists were “assured of certain certainties” and felt that essentially all the basic aspects of physics had been elucidated and that “our future discoveries must be looked for in the sixth place of decimals” (48). Then came quantum mechanics which changed everything. In a similar fashion, many in the hematopoietic stem cell field appear to feel that the stem cell is close to final definition with progressive progress in purification and that next steps simply are going to the sixth place of decimals. Rather, we think that we are entering the area of quantum stemonics where a true understanding of stem cell biology beckons.

## Conflict of Interest Statement

The authors declare that the research was conducted in the absence of any commercial or financial relationships that could be construed as a potential conflict of interest.
